# A Retrospective Analysis of Rituximab Treatment for B Cell Depletion in Different Pediatric Indications

**DOI:** 10.3389/fped.2021.651323

**Published:** 2021-11-30

**Authors:** Merlin Wennmann, Simone Kathemann, Kristina Kampmann, Sinja Ohlsson, Anja Büscher, Dirk Holzinger, Adela Della Marina, Elke Lainka

**Affiliations:** ^1^Department of Pediatric Gastroenterology, Hepatology and Liver Transplantation, University Children's Hospital, Essen, Germany; ^2^Department of Pediatric Nephrology and Kidney Transplantation, University Children's Hospital, Essen, Germany; ^3^Department of Pediatric Hematology-Oncology, University Children's Hospital, Essen, Germany; ^4^Department of Neuropediatrics, Developmental Neurology and Social Pediatrics, University Children's Hospital, Essen, Germany

**Keywords:** rituximab, B cell depletion, hypogammaglobulinaemia, immunodeficiency, antibody, pediatric

## Abstract

**Background:** Rituximab (RTX) is used in cancer therapy as well as in the treatment of autoimmune diseases and alloimmune responses after transplantation. It depletes the disease-causing B cells by binding to the CD (cluster of differentiation) 20 antigen. We evaluate different pediatric treatment protocols (via fixed treatment schedule, B cell- or symptom-controlled) and their therapeutic effects.

**Methods:** Demographic information, clinical and laboratory characteristics, and special laboratory values such as immunoglobulin G (IgG), CD19 positive B cells and Epstein-Barr viral load were retrospectively analyzed in children treated with RTX between 2008 and 2016.

**Results:** Seventy-six patients aged 1 to 19 (median 13) years were treated with 259 RTX infusions. The spectrum of diseases was very heterogeneous. RTX led to a complete depletion of the B cells. The reconstitution time varied between patients and was dependent on the application schedule (median 11.8 months). Fourteen out of 27 (52%) patients developed hypogammaglobulinaemia. The risk of IgG deficiency was 2.6 times higher in children under 4 years of age than in olderones. In the last group IgG deficiency developed in only 38% of the cases (*n* = 8). Recurrent and severe infections were observed each in 11/72 (15%) patients. Treatment-related reactions occurred in 24/76 (32%) cases; however, treatment had to be discontinued in only 1 case. In 16/25 (76%), the Epstein-Barr viral load dropped below the detection limit after the first RTX infusion.

**Conclusion:** RTX is an effective and well-tolerated drug for the treatment of oncological diseases as well as autoimmune and alloimmune conditions in children. B cell depletion and reconstitution varies both intra- und interindividually, suggesting that symptom-oriented and B cell-controlled therapy may be favorable. Treatment-related reactions, IgG deficiency and infections must be taken into account.

## Background

Rituximab (RTX) is a genetically engineered chimeric antibody. The variable region, which is directed against the CD (cluster of differentiation) 20 surface molecule, consists of murine light and heavy chain sequences. The constant region corresponds to of human immunoglobulin G1. The constant region activates the body's own immune response by triggering processes such as complement activation, killer cell recruitment and apoptosis. This leads to a decrease in the number of B cells in the peripheral blood. This process is known as B cell depletion and is used for management of the therapy. Since 2020, RTX is licensed for use in children with granulomatosis with polyangiitis, microscopic polyangiitis and non-Hodgkin lymphoma. There remains uncertainty about dosage, effectiveness and safety in treating children (1). Clinical trials in adults suggest that RTX is indicated for the treatment of conditions causing a humoral immune response. Unlicensed use of medication in pediatrics is widespread in Europe: at least 50% of drugs used in children have no approval for this age group. This increases to over 90% in pediatric intensive care medicine and neonatology (2). We present clinical characteristics, laboratory parameters, effectiveness and safety data in children with a variety of diseases receiving RTX in a German University Children's Hospital with vast experiences in organ and bone marrow transplantation, pediatric immunology and pharmacology.

## Materials and Methods

### Clinical Data Collection

All patients treated with RTX in the University Children's Hospital Essen between 2008 and 2016 were included in this retrospective analysis. Demographic information, clinical and laboratory characteristics, and special laboratory values such as immunoglobulin G (IgG), CD (cluster of differentiation) 19 positive B cells and Epstein-Barr virus (EBV) viral load were collected and analyzed using electronic and paper-based patient records.

### Treatment With RTX and Monitoring

RTX (MabThera^®^) was administered as an intravenous (i.v.) infusion at a dose of 375 mg/m^2^ body surface area over 4 h in an inpatient setting. Flow rate of RTX was increased according to a defined protocol (Supplementary Table 1).

Premedication (i.v.) with an antipyretic drug (10 mg/kg acetaminophen), an antihistamine (0.02–0.03 mg/kg clemastine) and, depending on protocol, prednisolone (1–2 mg/kg) was administered to reduce the risk of treatment-related reactions (TRR). In case of a hypersensitivity reaction to RTX, the infusion was paused and emergency medication consisting of 0.025 mg/kg clemastine and 2 mg/kg prednisolone was administered. Treatment was re-started once symptoms had resolved. The patients' vital signs were closely monitored. Co-trimoxazole (trimethoprim/sulfamethoxazole) was given as a prophylaxis for bacterial infection with pneumocystis jirovecii and amphotericin B as prophylaxis for fungal infections, depending on the underlying disease.

The number and intervals of RTX infusions varied depending on the underlying disease. For the treatment of post-transplant lymphoproliferative disorder (PTLD) and oncological diseases an individual fixed therapy scheme was used. Other children were either treated via B cell control (i.e., the next dose was administered after B cells were regenerated up to 10%) or according to clinical and laboratory findings associated with disease activity.

Age-specific value ranges for IgG (g/l) from the laboratory database of the University Hospital Essen were used to assess for hypogammaglobulinemia. CD20 receptors are internalized after binding to RTX and the proportion of CD19 positive B cells (%) was used as an indirect marker for treatment effect. The qualitative and quantitative detection of EBV-PCR (IU/ml) was carried out by polymerase chain reaction (PCR). The following laboratory parameters were recorded as part of clinical routine before and 3 months after each dose: blood cell count (n/l), differential blood count (n/l), hemoglobin concentration (g/dl), transaminases (U/l), C-reactive protein (CRP, mg/dl), lactate dehydrogenase (LDH, U/l), and creatinine (mg/dl).

### Statistical Analysis and Ethics Commission

Descriptive analyses included mean and standard deviation for normal distribution, median and range for non-normal distribution as well as absolute and relative frequencies for categorical data. Anonymized data was analyzed using Microsoft Excel 2016 and IBM SPSS Statistics Version 25. The following statistical tests were used: Paired *T*-test, Wilcoxon test and Fisher's exact test. To test hypotheses, the significance level was set to α = 10%.

This retrospective analysis was approved by the ethics committee of the Medical Faculty of the University Duisburg-Essen (vote 16-7140-BO).

## Results

### Patient Cohort

Seventy-six children aged 1 to 19 years (median 13 years, female 35, male 41) were treated with RTX for various diseases. The median observation period was 47.5 (17.6–114) months (Table 1). Other diseases (*n* = 8) were:

° EBV-induced hepatitis/X-MEN-syndrome (1)
° EBV-associated hemophagocytic syndrome (1)
° EBV-induced hepatitis with hemophagocytic syndrome (1)
° EBV infection after allogenic bone marrow transplantation (1)
° Increasing EBV viral load after allogenic bone marrow transplantation (1)
° EBV reactivation after allogenic bone marrow transplantation (2)
° Pseudotumor orbitae (1)

**Table 1:** Patient cohort and different RTX regimes (*n* = 76).

**Disease category**	**Patients**	**RTX interval**	
		**1-4 weeks**	**>4 weeks**
**Post-transplant lymphoproliferative disorder(5 after liver, 5 after kidney, 2 after bone marrow transplantation)**	**12**	**11**	**1**
*Monomorphic (Burkitt lymphoma or diffuse large-cell B cell lymphoma)*	9		
*Polymorphic*	2		
*Unknown*	1		
**Organ transplant rejection(7 humoral, 1 cellular, 5 mixed cellular and humoral)**	**13**	**0**	**13**
*Liver transplantation*	3		
*Kidney transplantation*	10		
**Autoimmune diseases**	**24**	**5**	**19**
*Juvenile dermatomyositis*	2		
*Granulomatosis with polyangiitis*	3		
*Juvenile myasthenia gravis*	3		
*Systemic lupus erythematosus with Shrinking lung disease*	1		
*Atypical hemolytic uremic syndrome*	2		
*Thrombotic-thrombocytopenic purpura*	1		
*Anti-N-methyl-D-aspartate receptor (NMDAR)-encephalitis*	1		
*Membrano-proliferative glomerulonephritis type 1*	1		
*Membrano-proliferative glomerulonephritis type 2*	1		
*Microscopic polyangiitis*	1		
*Immunological disease associated with membrano-proliferativeglomerulonephritis type 1*	1		
*Haemophilia with inhibitors to factor VIII*	1		
***Autoimmune hepatitis***	**6**		
**Oncological diseases**	**6**	**5**	**1**
*Burkitt leukemia*	2		
*Acute lymphatic leukemia*	1		
*Mediastinal large B cell lymphoma*	1		
*Diffuse large centroblastic B cell lymphoma*	1		
*Unknown cervical lymphadenopathy*	1		
**Nephrotic syndrome**	**13**	**0**	**13**
*Minimal-change glomerulonephritis (7 steroid-dependent, 2 steroid-sensitive)*	9		
*Focal-segmental glomerulosclerosis (1 steroid-resistant)*	1		
*Unknown*	3		
Other diseases*	**8**	**2**	**6**

* *Other diseases (n = 8) were*.

### Number of Applications and Intervals of RTX

**Figure 1:**
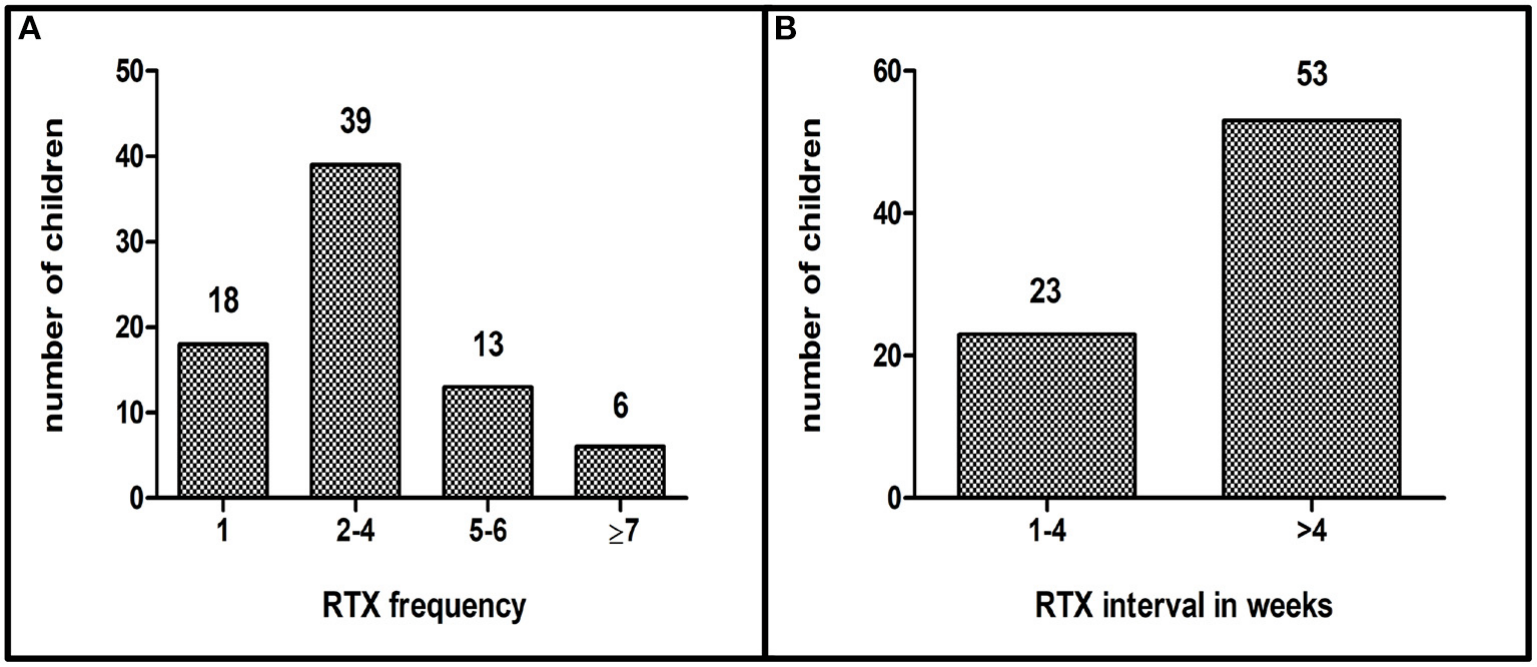
**(A)**: Application frequency of RTX, **(B)**: Application intervals of RTX in weeks (*n* = 76).

The number and intervals of the RTX infusions varied depending on the underlying disease. Patients were classified according to number of application (1 infusion *n* = 18, 2–4 infusions *n* = 39, 5–6 infusions *n* = 13, 7 and more infusions *n* = 6) and application interval (group 1: 3–6 infusions with short interval (1–4 weeks) *n* = 23, group 2: infusions with long intervals (>4 weeks) *n* = 53 (Figures 1A,B; Table 2).

**Table 2:** RTX frequency and RTX intervals according to underlying disease (*n* = 76); PTLD = post-transplant lymphoproliferative disorder.

	**RTX frequency**									**RTX intervals**	
	**1**	**2**	**3**	**4**	**5**	**6**	**7**	**8**	**9**	**1–4 weeks**	**>4 weeks**
PTLD	0	1	2	0	1	8	0	0	0	11	1
Rejection	9	3	1	0	0	0	0	0	0	0	13
Autoimmune diseases	5	6	2	7	0	2	1	1	0	5	19
Oncological diseases	2	0	0	2	0	1	0	1	0	5	1
Other diseases	2	2	1	3	0	0	0	0	0	2	6
Nephrotic syndrome	0	3	3	3	1	0	1	1	1	0	13
Number of patients *n* =	18	15	9	15	2	11	2	3	1	23	53

### B Cell Suppression and Reconstitution

**Figure 2:**
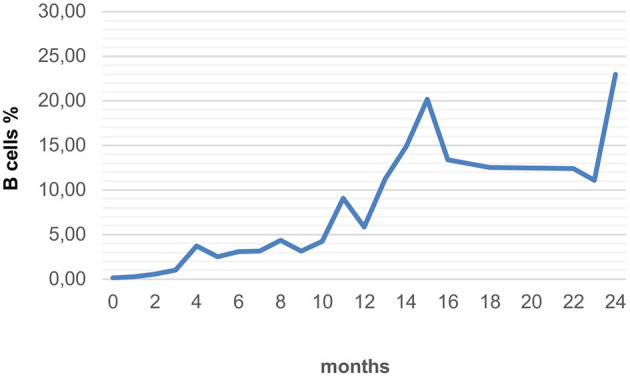
Average recovery of B cells in all forms of RTX applications with long-term monitoring (*n* = 44).

CD19 positive B cells were recorded up to 24 months after the last RTX application in only 44/76 (58%) patients because laboratory values like CD19 positive B cells were not routinely assessed (Figure 2).

Group 1 (*n* = 23) included patients who received RTX 3–6 times at short intervals (1–4 weeks). The median age of children with PTLD, autoimmune and oncological diseases and was 8.8 (1–18) years. In the period from 1 to 4 weeks after the last treatment, the median proportion of B cells was 0.04% (*n* = 4). The B cells reached 5% (*n* = 4) after a median time of 6.5 months and 10% (*n* = 3) after 15 months. There is a wide time range, from 3 to 14 months for the 5% mark, and from 6 to 20 months for the 10% mark. In 2 patients, B cells had not increased above 5% after 1 and 2 years.

Group 2 (*n* = 53) included patients with multiple infusions of RTX at intervals of several months. The median age of children with autoimmune hepatitis, rejection reactions after organ transplantation and nephrotic syndrome was 11.7 (1–19) years. RTX depleted the B cells reliably and almost completely. The median percentage of B cells in the blood count 1–4 weeks after the first infusion was 0.09% (*n* = 34). B cells increased to 5% (*n* = 13) after a median time of 5 months and to 10% (*n* = 8) after 8.5 months. The amount of B cells remained below 5% even 1 year after treatment in one 15-year-old patient with autoimmunhepatitis. Interindividual heterogeneity of B cell reconstitution after the first dose of RTX as well as intraindividual differences in the reconstitution time were measured. In 6 patients, the time taken to reach the 5% mark after the 1st and 2nd RTX administration were compared: In 3 out of 6 patients, the reconstitution times after the 1st and 2nd administration were the same (± 1 month). In one patient it took 2.5 months longer, two others recovered 2 or 7 months faster than after the first dose.

### Hypogammaglobulinaemia

Fourteen out of 27 (52%) of patients developed hypogammaglobulinaemia during treatment with RTX. Only 1 baby with PTLD after liver transplantation due to biliary atresia had a persistent hypogammaglobulinemia requiring IgG substitution after her first RTX infusion. Because of the PTLD she received 6 RTX infusions during a short application interval. The other 13 children had a transient hypogammaglobulinemia.

All patients younger than 4 years old (*n* = 6) developed hypogammaglobulinaemia. Among the 21 patients between 4 and 19 years of age, IgG deficiency developed in 8 (38%). In children under 4 years of age, the risk of hypogammaglobulinaemia was significantly higher (2.6 times) than in patients aged 4–19 years old (*p* = 0.016).

**Figure 3:**
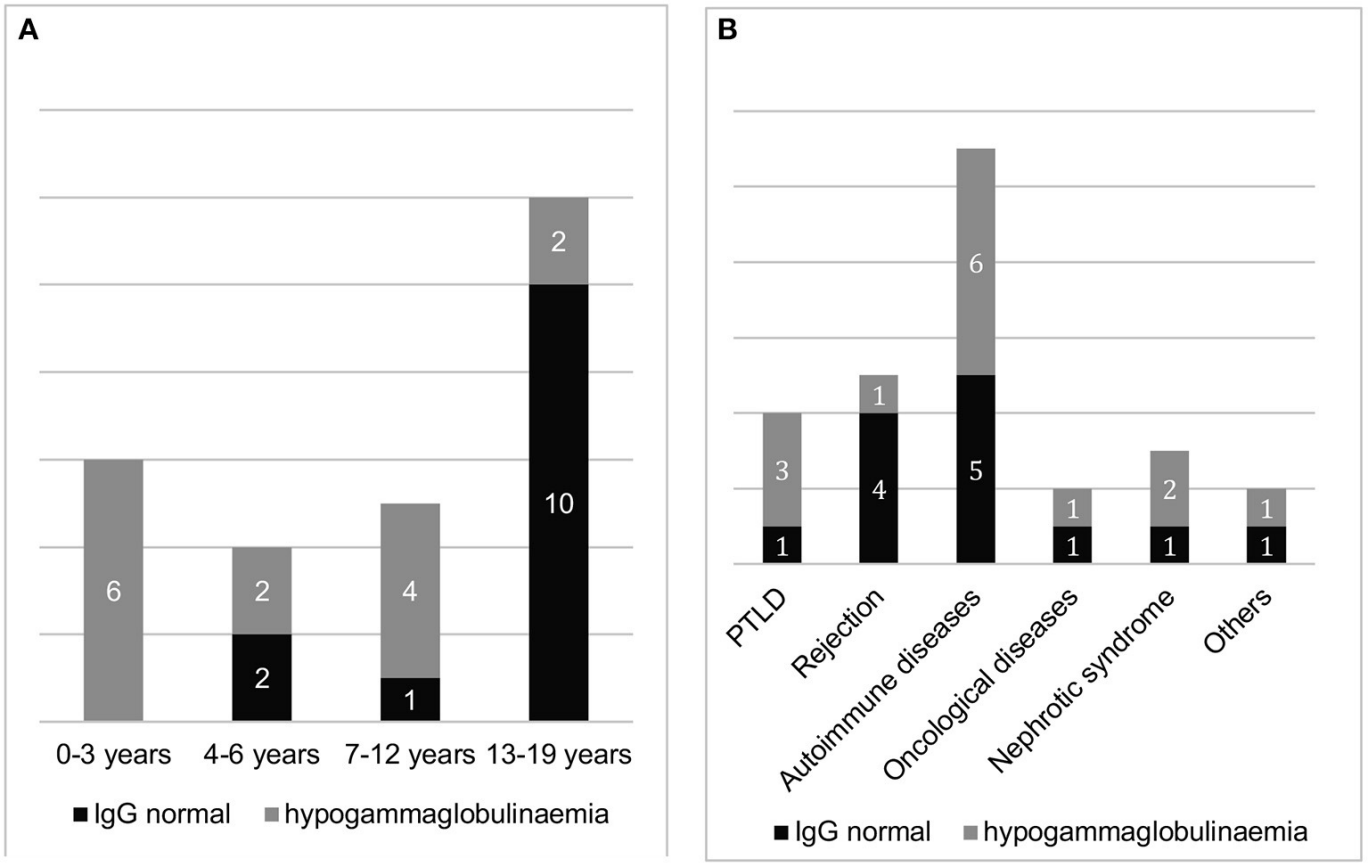
Proportion of patients with and without hypogammaglobulinaemia after RTX treatment (with previously normal IgG levels), **(A)**: sorted by age group and **(B)**: sorted by underlying disease (*n* = 27).

The probability of occurrence of hypogammaglobulinaemia assessed as a function of the number of RTX infusions is 23% with only 1 treatment (*n* = 13) and 79% with more than 3 infusions (*n* = 14). The risk is significantly (3.4 times) higher in the latter group (*p* = 0.007). While the likelihood of developing hypogammaglobulinemia was thus significantly related to the number of infusions and the age of patients, we found no significant statistical correlation between IgG deficiency and the treatment interval at 3–6 infusions or the disease group (Figures 3A,B).

### Infections

Antibacterial and antifungal prophylaxis was dependent on the protocol used and underlying disease. Sixty out of 76 (79%) patients received pneumocystis jirovecii-pneumonia (PJP) prophylaxis in the form of the oral antibiotic co-trimoxazole. To prevent fungal infections, 28/76 (37%) patients received oral amphotericin B. No infection with PJP developed in either the group with co-trimoxazole prophylaxis (60/76) or the group without prophylaxis (16/76). There was no thrush or another fungal infection recorded in the groups with (28/76) or without antifungal prophylaxis (48/76).

In 11/72 (15%) patients, recurrent infections occurred in the form of gastrointestinal infection, bronchitis, pneumonia, sepsis, peritonitis, urinary tract infection, pyelonephritis, otitis media or varicella zoster virus infection. Six out of these 11 and 5 other patients [a total of 11/72 (15%), female 3, male 8, median age 2 (1–8) years] also developed severe infections in the form of sepsis, pneumonia or a severe/prolonged urinary tract infection. Respiratory/urinary tract infections and otitis media were often caused by Pseudomonas aeruginosa; Clostridia could often be detected in gastrointestinal infections. More than 50% of severe infections affected young children under PTLD treatment, i.e., 3 RTX infusions once a week followed by 3 RTX infusions every 3 weeks according to PTLD protocol.

### Epstein-Barr Virus PCR

Before RTX administration, EBV-PCR was detectable in the blood of 25 patients. In 16/25 (76%) patients EBV viral load dropped below the detection limit after the first RTX infusion. In 5 patients the virus remained detectable afterwards but with reduced viral load in 4 patients; in 4 others no data are available. However, after a single administration of RTX, the EBV viral load in the blood of most patients dropped.

### Treatment-Related Reactions

**Figure 4:**
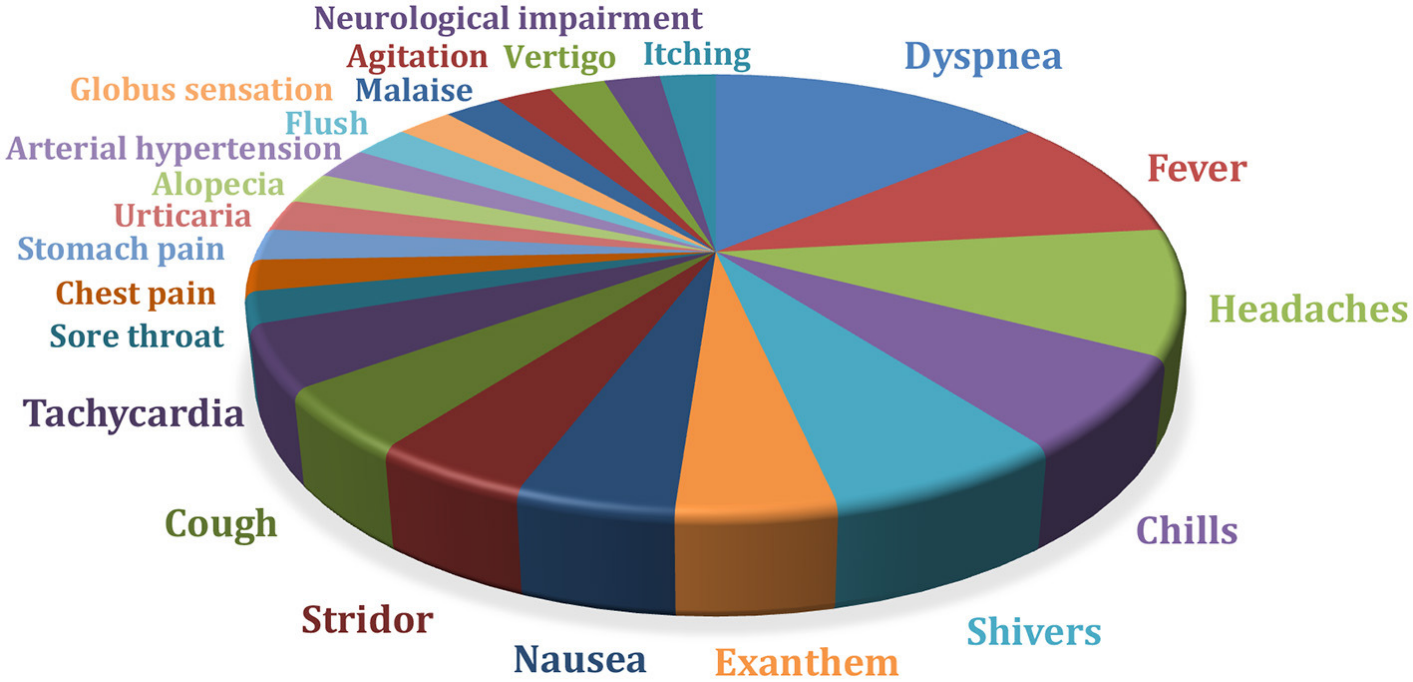
TRR, treatment-related reactions in a total of 259 infusions administered in n=76 patients (in some cases several TRR occurred per infusion) (*n* = 76); neurological impairment = aphasia, hypersalivation, tingling paresthesias.

A total of 259 infusions were analyzed. TRR occurred about every 10th dose. Twenty-four out of 76 (31.6%) patients had TRR during or shortly after the infusion of RTX. Most frequently, the patients complained of dyspnoea, chills, shivers, headaches, or fever (Figure 4). In 15/24 (62%) patients TRR occurred during the first dose. In the other patients, intolerance reactions only became apparent in the course of the following infusions. In the case of further infusions, these could also be absent. Emergency medication (prednisolone, clemastine) was applied in 6/76 (8%) patients. In one child RTX was stopped due to TRR.

## Discussion

We present the clinical characteritics, laboratory parameters, effectiveness, and safety risks of children receiving RTX. In our heterogeneous group RTX (MabThera^®^) was administered either once or several times over a short period of time or for many months or years. Short application intervals were recommended in fixed therapy protocols (PTLD, oncological diseases) or with simultaneous plasmapheresis (*n* = 25, autoimmunhepatitis, rejection reaction after organ transplantation or other autoimmune diseases). Two children, one with recurrence of Progressive familial intrahepatic cholestasis 2 after liver transplantation and one with Juvenile myasthenia gravis, received immunadsorption. Longer infusion intervals were used mostly with B cell- or symptom-controlled therapy regimes (e.g., nephrotic syndrome). With RTX treatment most patients led to improvement of laboratory findings and clinical presentation. Due to the severity of the underlying disease and necessity for additional immunosuppression, plasmapheresis, therapeutic IgG substitution and symptom-oriented interventions, our data do not assess the effects of RTX alone. Therefore, we concentrate on selected laboratory aspects and side-effects.

### B Cell Suppression and B Cell Reconstitution

Treatment with RTX leads to B cell depletion and therefore a reduced antibody formation as well as suppression of the B cell-T cell interaction (3). Previous assessments of the treatment of nephrotic syndrome have indicated a correlation between dose and application interval on the one hand and B cell reconstitution time and recurrence of disease activity on the other (4). The higher the initial dose, the longer the B cell reconstitution takes and the later a relapse in disease activity occurs (4, 5). However, relapses do not always occur depending on the level of B cells. RTX affects not only the number of B cells, but also the function of T cells, including regulatory T cells (T-reg) (6). A decrease in this cell population is associated with disease recurrences. Conversely, an increase can be observed during the clinical remission (7). RTX increases the number and function of T-reg cells and thus contribute to induction of disease remission (8). Sellier-Leclerc et al. reported long-lasting maintenance of disease remission through RTX, even after B cell reconstitution (9). Ongoing remission through permanent B cell suppression could be achieved in patients included in our analysis. Therefore, B cell reconstitution seems to be a good but not the only prognostic marker for the occurrence of relapses.

We found B cell reconstitution time varies intra- and interindividual. B cells should be measured before the next planned RTX infusion. In a few cases there can be a significantly prolonged B cell depletion independent of age, disease group and application frequency (10). B cells in children under 10 years of age recover faster than in children over 10 years of age. Influencing variables are underlying disease groups and co-medication (e.g., chemotherapy) (9).

### Hypogammaglobulinaemia

Hypogammaglobulinaemia during RTX therapy is a consequence of inadequate reconstitution of the B cells (11). Risk factors for its development are a high dose of RTX, pre-existing low immunoglobulin levels or additional treatment with mycophenolate-mofetil, purine analogs (12), or cyclophosphamide (13). In some cases, hypogammaglobulinaemia can persist for many years after treatment (14). IgG production takes place in the long-lived plasma cells located in the bone marrow and other lymphatic organs and is therefore difficult to measure. There are no meaningful data on their age-dependent quantitative presence in the body.

In the current study, 50% of all patients developed at least intermittent hypogammaglobulinaemia. The more often a patient received RTX, the higher was the risk of hypogammaglobulinemia. The probability of developing hypogammaglobulinaemia was highest in children under 4 years of age. Pre-existing hypogammaglobulinaemia worsened under RTX therapy. Cumulative dose and the dose interval are considered risk factors for the development of opportunistic infections (15). In one adult study, 4–4.5% received immunoglobulin substitution following RTX. Among these patients, a higher cumulative immunoglobulin replacement dose was associated with a reduced risk of complications (16). Measurement of IgG levels before and after administration is useful. If necessary, immunoglobulin substitution (400–500 mg/kg/month) should be initiated. There can be a prolonged requirement for IgG replacement therapy, but also IgG recovery is reported (17).

### Infections

RTX interferes with the humoral immune system and can increase the risk of bacteremia, sepsis, pneumonia, and other opportunistic infections. Type and frequency of infections hardly differ between adults and children (18). None of the patients in this observation cohort developed PJP or mycosis, regardless of whether or not they underwent prophylaxis with co-trimoxazole or oral amphotericin B. Not using the latter (very unpalatable) substance would improve the patient's quality of life. The need for thrush prophylaxis with oral amphotericin B depends on risk factors like neutropenia, intensive RTX protocol and mycosis in the past medical history. Only few cases of fungal infections caused by RTX have been reported in the literature (19). The data of a meta-analysis from 2015 showed that the use of RTX is associated with a significantly higher risk of developing PJP (20). PJP prophylaxis has been shown to be effective to prevent this infection. We would recommend, co-trimoxazole should be taken for at least 3 months after RTX administration depending on the underlying disease and associated co-medication.

In our study, 15% of all patients developed recurrent infections, and 15% severe infections. During RTX treatment about over 30% of patients developed infections, mostly respiratory, skin and soft tissue infections and pyelonephritis (21). Risk factors for infections are co-medication, different treatment plans for RTX, plasmapheresis and immunadsorption, low IgG levels (for more than 6 months), chronic lung disease, and cardiac insufficiency (12, 22). Due to retrospective data analysis, also in our cohort, we cannot differ whether the infections are caused by RTX alone or due to the co-medication.

### Epstein-Barr Virus

The drop in the viral load during our RTX treatment probably occurs because the replication of the EBV mainly takes place in B lymphocytes and RTX leads to a depletion of a B cell subpopulation. A median EBV suppression period of 8 months is described after a single administration (23). RTX is recommended as a therapy for fulminant EBV infection (24). RTX-induced B cell depletion before transplantation can prevent the spread of EBV from the donor organ to the recipient. Three out of 5 patients who received RTX 30 days prior to kidney transplantation remained EBV negative after transplantation (25). Preemptive therapy containing RTX is expected to reduce the incidence of PTLD after hematopoietic stem cell transplantation and to improve post-transplantation outcomes in children (26).

### Treatment-Related Reactions

Cytokines, which are responsible for the observable symptoms, are released via different signaling pathways leading to sometimes severe TRR. The release of tumor necrosis factor-alpha and interleukin-6 was observed early on (27). A high lymphocyte count appears to predispose to TRR. The probability of occurrence of TRR also appears to depend on the patient's underlying disease and co-medication. Patients with nephrotic syndrome are less affected than those treated for lymphoma (28).

If side effects occur, a pause and subsequent continuation of the infusion with a lower flow rate and, if necessary, symptom-oriented pharmacotherapy have proven to be effective. In our cohort, emergency medication had to be used in 8% of patients. In only one child, TRR were the reason for stopping RTX infusion, so we conclude that RTX is save in most children.

### Limitations of Our Study

Limitations of our analysis are varying quality of data documentation, heterogeneity of patients, different disease groups, as well as the retrospective study character. Laboratory values like CD19 positive B cells, IgG and EBV viral load were not always measured because these parameters are usually not part of clinical routine. Only afterwards, a protocol for monitoring of RTX treatment has been established based on clinical experience (Supplementary Table 1). Interaction of RTX with concomitant therapy could not be analyzed in detail, and the variable duration of follow-up may have had an impact on the recorded treatment and outcome. The comparability is therefore limited, although our inhomogeneous study group reflects our daily challenge.

## Conclusion

RTX has proven to be an effective and well-tolerated drug for the treatment of oncological diseases as well as autoimmune and alloimmune conditions in children. B cell depletion and reconstitution varies intra- und interindividually, suggesting that symptom-oriented and B cell-controlled therapy may be favorable. Treatment-related reactions, IgG deficiency and infections must be taken into account. Further studies based on more standardized datasets are mandatory, in particular for comparison of different treatment strategies.

## Data Availability Statement

The datasets analyzed during the current study are available from the corresponding author on reasonable request.

## Ethics Statement

The studies involving human participants were reviewed and approved by Ethic Committee of the Medical faculty of the University Duisburg-Essen (vote 16-7140-BO). Written informed consent from the participants' legal guardian/next of kin was not required to participate in this study in accordance with the national legislation and the institutional requirements.

## Author Contributions

MW and EL contributed to the study conception and design. Data collection and analysis were performed by MW. The first draft of the manuscript was written by MW and EL. All authors commented on previous versions of the manuscript, read and approved the final manuscript.

## Conflict of Interest

The authors declare that the research was conducted in the absence of any commercial or financial relationships that could be construed as a potential conflict of interest.

## Publisher's Note

All claims expressed in this article are solely those of the authors and do not necessarily represent those of their affiliated organizations, or those of the publisher, the editors and the reviewers. Any product that may be evaluated in this article, or claim that may be made by its manufacturer, is not guaranteed or endorsed by the publisher.

## Abbreviations

CD: Cluster of differentiation
CRP: C-reactive protein
EBV: Epstein-Barr virus
IgG: Immunglobulin G
i.v.: Intravenous
LDH: Lactate dehydrogenase
PCR: Polymerase chain reaction
PJP: Pneumocystis jirovecii-pneumonia
PTLD: Post-transplant lymphoproliferative disorder
RTX: Rituximab
T-reg: Regulatory T cells
TRR: Treatment-related reactions.
