# Supplementary material for: A Retrospective Analysis of Rituximab Treatment for B Cell Depletion in Different Pediatric Indications
Source: Front Pediatr. 2021 Nov 30;9:651323. doi: 10.3389/fped.2021.651323 (PMC8669827; doi:10.3389/fped.2021.651323)
Supplement: Supplementary file 1 [file Table_1.DOCX]

**Therapy scheme for the University Children's Hospital Essen**

Surname: First name: Date of birth: Weight:

**Symptoms: Abnormal examination results:**

**Infections: Medication:**

**Live vaccinations (≤ 4 weeks):**

**Abnormal laboratory values:**

CRP 🞎 Complete blood count 🞎 Liver enzymes 🞎 Bilirubin (total) 🞎

Coagulation 🞎 Blood gas analysis 🞎 Electrolytes 🞎 CD19/20 🞎

IgG 🞎

**Did you provide information about off-label use and side effects?**

**Signed declaration of consent?**

**Laboratory controls:**

Before each infusion: blood count / differential blood count, kidney parameters, coagulation, CRP, transaminases, electrolytes, IgG.

Before the first infusion: exclude EBV-, CMV-, HIV-, hepatitis B/C- and tuberculosis-infection,

check the immunogram, determinate the vaccination titre and check for missing vaccinations.

During therapy: EBV-PCR, IgG, CD19-positive B cells.

**Medical history prior to therapy:**

Cardiovascular disease? Allergies? Infections? Vaccinations with live vaccines in the last 4 weeks? Vaccination status? Concomitant medication? Chest pain? Fever? Skin and mucous membrane changes? Abdominal pain? Stool abnormalities? Neurological abnormalities? Contraception? Pregnancy must be prevented during therapy and it must be safely prevented for 6 months afterwards!

**Side effects:**

Fever, chills, dyspnea, headache, rigor, rash, fatigue, cardiac events. Infusion reactions occur a few minutes and up to 24 hours after rituximab was administered.

They are not to be confused with a cytokine release syndrome / tumor lysis syndrome (occurrence 1 - 2 hours after infusion). Delayed allergic reactions can rarely occur within the first 2 weeks after administration, e.g. exanthema and fever.

Neutrocytopenia, leukocytopenia and thrombocytopenia, viral, bacterial, fungal and herpes infections, reactivation of hepatitis B, bronchitis, psychological reactions (depression, insomnia), progressive multifocal leukoencephalopathy, paresthesia, vasodilatation, hyper- and hypotension, nausea, vomiting, musculoskeletal pain, common cold, fatigue syndrome, multiple organ failure.

**Onset of drug effect:** after 2 - 12 weeks.

**Duration of B-cell depletion:** approx. 6 months, different reconstitution times are possible!

**Premedication:**

Clemastine 0,03 mg/kg body weight i. v.

Prednisolone 2 mg/kg body weight i. v.

Paracetamol 10-15 mg/kg body weight p. o. (max. 1000 mg)

**Prophylaxis of opportunistic infections:**

Antifungal prophylaxis (Candida) with Ampho-Moronal® (4 x 1 - 2 ml oral drops or 4 x 1 lozenges after meals) in the first 4 weeks after rituximab infusion. Continuation of prophylaxis for at least 3 months after the last infusion in case of prolonged B-cell depletion and high risk of infection (e.g. in the case of leukopenia) or a previous fungal infection.

Infection prophylaxis (P. jirovecii) with cotrimoxazole (5 mg / kg body weight / day in 2 doses from Friday to Sunday) for 3 months after the last infusion.

In case of hypogammaglobulinaemia, if necessary, IgG substitution s. c. or i. v.!

**Place of administration:**

In the infirmary or in a monitoring room of the ambulance. Plan the first three doses in the infirmary. Keep monitoring of the vital parameters. Keep emergency medication prepared!

**In case of side effects during administration:**

Slow down the infusion rate, re-administer clemastine 0,03 mg / kg body weight i. v. and prednisolone 2 mg / kg body weight i. v.

**Dosage and infusion rate:**

375 mg/m^2^ body surface area.

Application over approx. 4 hours. Test dose over 15 minutes (10 ml).

< 550 mg rituximab in 250 ml NaCl 0,9%: 30 ml in the first, 60 ml from the second hour;

≥ 550 mg rituximab in 500 ml NaCl 0,9%: 50 ml in the first, 100 ml in the second, 150 ml from the third hour.

After that, application of isotonic electrolyte solution (10-20 ml / kg body weight over 1 hour i. v.).

| Time | Temp. °C | RR  mm/Hg | HR /  min | BR /  min | SaO_2 %_ | **ml / h** | Side effects | Signature |
| --- | --- | --- | --- | --- | --- | --- | --- | --- |
| 0 min |  |  |  |  |  |  |  |  |
| 15 min |  |  |  |  |  |  |  |  |
| 30 min |  |  |  |  |  |  |  |  |
| 60 min |  |  |  |  |  |  |  |  |
| 120 min |  |  |  |  |  |  |  |  |
| 180 min |  |  |  |  |  |  |  |  |
| 240 min |  |  |  |  |  |  |  |  |
| 300 min |  |  |  |  |  |  |  |  |
